# Differentiating Exposure From Consequence: A Longitudinal Examination of Trauma and Posttraumatic Distress in a Public HIV Care Clinic

**DOI:** 10.1007/s10461-026-05089-3

**Published:** 2026-03-14

**Authors:** Norik Kirakosian, Yumei O. Chen, Maria M. Llabre, Hanna Vasconcello, Allan Rodriguez, Steven A. Safren

**Affiliations:** 1 Department of Psychology, University of Miami, 5665 Ponce De Leon Blvd, Coral Gables, Florida 33146-2510 USA; 2 Department of Medicine, University of Miami Miller School of Medicine, Miami, FL USA

**Keywords:** HIV, Trauma, Posttraumatic stress, Longitudinal, Antiretroviral adherence

## Abstract

Persons living with HIV (PLWH) are disproportionately exposed to traumatic events, which are associated with posttraumatic psychological distress and impaired HIV-related health outcomes (e.g., antiretroviral non-adherence, HIV viremia, lower CD4 count). Prior literature has not adequately differentiated the effects of trauma and posttraumatic distress on HIV-related health outcomes. This study examined longitudinal data of trauma exposure and posttraumatic psychological distress among PLWH. Participants were 255 PLWH in care at a public HIV clinic that completed baseline and one-year follow-up psychosocial assessments between April 2017 and May 2024. Assessments included measures of lifetime trauma exposure, symptoms of depression, anxiety, and posttraumatic distress, and antiretroviral adherence. HIV RNA viral load and CD4 count were extracted from medical records. Factor analysis was used to consider multifaceted posttraumatic responses. Path analysis was used to examine direct and indirect effects of trauma exposure and posttraumatic distress on HIV-related outcomes. Most participants endorsed lifetime trauma exposure (88.2%). Trauma exposure was associated with increased posttraumatic psychological distress (est.=0.281, *p* = 0.004). Psychological distress predicted lower antiretroviral adherence (est.=-0.070, *p* = 0.033), which was associated with higher log HIV RNA viral load (est.=-0.477, *p* < 0.001) and lower CD4 count (est.=84.754, *p* < 0.001). Controlling for distress, trauma exposure did not predict HIV-related health outcomes. Trauma exposure was highly prevalent and posttraumatic psychological distress predicted poorer HIV-related health outcomes at follow-up. These findings highlight the need for universal trauma-informed care and the utility of integrated psychological services for those impacted by posttraumatic distress within HIV treatment settings to improve HIV-related health outcomes.

## Resumen

Las personas que viven con el VIH (PVVIH) están expuestas de manera desproporcionada a experiencias traumáticas, que se asocian con angustia psicológica postraumática y peores resultados de salud relacionados con el VIH (por ejemplo, falta de adherencia al tratamiento antirretroviral, viremia del VIH, recuento más bajo de CD4). La literatura anterior no ha diferenciado adecuadamente los efectos del trauma y el malestar postraumático en los resultados de salud relacionados con el VIH. Este estudio examinó datos longitudinales de exposición al trauma y malestar psicológico postraumático entre las PVVIH. Los participantes fueron 255 personas que viven con el VIH atendidas en una clínica pública especializada en VIH, que completaron evaluaciones psicosociales iniciales y de seguimiento al cabo de un año entre abril de 2017 y mayo de 2024. Las evaluaciones incluyeron medidas de exposición al trauma a lo largo de la vida, síntomas de depresión, ansiedad y angustia postraumática, y adherencia al tratamiento antirretroviral. La carga viral del ARN del VIH y el recuento de CD4 se extrajeron de los registros médicos. Se utilizó el análisis factorial para considerar las respuestas postraumáticas multifacéticas. Se utilizó el análisis de trayectoria para examinar los efectos directos e indirectos de la exposición al trauma y la angustia postraumática en los resultados relacionados con el VIH. La mayoría de los participantes confirmaron haber estado expuestos a traumas a lo largo de su vida (88.2%). La exposición a traumas se asoció con un aumento del malestar psicológico postraumático (est.=0.281, p=0.004). El malestar psicológico predijo una menor adherencia al tratamiento antirretroviral (est.=-0.070,* p*=0.033), lo que se asoció con una mayor carga viral del ARN del VIH (est.=-0.477, * p* < 0.001) y un recuento de CD4 más bajo (est.=84.754, * p* < 0.001). Controlar por angustia, la exposición al trauma no predijo los resultados de salud relacionados con el VIH. La exposición al trauma fue muy frecuente y el malestar psicológico postraumático predijo peores resultados de salud relacionados con el VIH en el seguimiento. Estos hallazgos ponen de relieve la necesidad de una atención universal informada sobre el trauma y la utilidad de los servicios psicológicos integrados para las personas afectadas por el malestar postraumático en los entornos de tratamiento del VIH, con el fin de mejorar los resultados de salud relacionados con el VIH.

## Introduction

Despite significant advancements in HIV prevention and care, persons living with HIV (PLWH) continue to be impacted by a disproportionate burden of trauma [1, 2]. Traumas are events beyond the normative experience that may be significantly distressing, life threatening, and/or permanently disabling, such as major natural or technological disasters, war and combat experiences, serious traffic accidents, significant family losses, and experiences of interpersonal violence [3]. PLWH report higher rates of exposure to trauma than the general population and are placed at higher risk for interpersonal victimization and sexual violence traumas in particular [1, 4–9]. Further, for many PLWH, HIV diagnosis and treatment may itself be experienced as traumatic, warranting special attention to remedying the consequences of trauma in this population [10–13].

Trauma has complicated the HIV treatment course since early in the global public health response [14–16]. Overall, PLWH with histories of trauma are more likely to experience suboptimal HIV-related health outcomes relative to trauma unexposed individuals [17–19]. Specifically, trauma interferes with medication adherence, appointment attendance, and other care engagement behaviors among PLWH [20–22]. Consequently, PLWH with trauma histories face greater challenges to achieving and sustaining viral suppression and immune health, with higher HIV RNA viral load and lower CD4 count [23–27].

Trauma may interfere with HIV-related health by placing PLWH at risk for psychological distress and impairment. The posttraumatic trajectory in the general population is characterized as a period of increased risk for mood and anxiety symptoms, as well as the emergence of depression, anxiety, and posttraumatic stress disorders [28–31]. Among PLWH, symptoms of depression, anxiety, and posttraumatic stress have been shown to impede HIV-related health behaviors such as medication adherence, and even suppress immune response and viral control via biological pathways [21, 32–41].

Although exposure to trauma is common, the psychological response to traumatic events is highly variable and may range from nonresponse and mild acute reactions to persistent and impairing psychological distress [42–44]. As such, not all PLWH exposed to trauma will experience distress, and not all of those with prior trauma histories and/or psychological reactions will experience HIV-related health impediments. However, the current state of the literature on the effects of trauma on HIV-related health outcomes has lacked a focus on differentiating trauma exposure from posttraumatic psychological response, conflating trauma exposure and posttraumatic distress. Existing studies have been limited by cross-sectional methods and by rarely assessing both exposure to trauma and posttraumatic psychological distress in the same models. In a prior cross-sectional study, our group showed that in the presence of both trauma exposure and symptoms of posttraumatic stress disorder, only the latter were associated with HIV medication adherence [20].

Using data from a primary care clinic in South Florida, a U.S. HIV epicenter, the present analysis aimed to differentiate the effects of trauma exposure from posttraumatic psychological distress with respect to HIV-related health outcomes. Building on our prior work [20], we used longitudinal path analysis within a structural equation modeling framework to examine the direct and indirect effects of trauma exposure and posttraumatic psychological distress on behavioral health outcomes (medication adherence) and biological health outcomes (HIV RNA viral load, CD4 count).

## Methods

### Participants and procedures

Study participants were PLWH receiving care at a public hospital in downtown Miami, Florida [45, 46]. We leveraged data from an ancillary clinic-based study focused on characterizing contextual and psychosocial characteristics among clinic patients. The clinic serves approximately 3,000 patients, all of whom were considered eligible for participation if they met these eligibility criteria: (1) being 18 years old and over, (2) receiving HIV care at the clinic, (3) able to speak and understand English, Spanish, and/or Haitian Creole, and (4) able and willing to provide informed consent. At the time of this analysis, 1,374 participants were enrolled between 2017 and 2023 and completed an interviewer-administered baseline assessment. Follow-up assessments were completed between 2020 and 2024. This was not a formal longitudinal study, and as such, follow-up assessments were added ad-hoc approximately two years after the initiation of the study, were offered optionally, were not systematically prioritized by recruitment staff, and were significantly limited in reach during COVID-related restrictions in the clinic. Therefore, our analytic sample included 255 participants (18.6% of the initial sample) that had completed two psychosocial assessments (M = 16.0 months apart). Plausible reasons for not completing follow-ups included loss to follow-up from the clinic (e.g., receiving care elsewhere), incarceration, death, pause of follow-ups during COVID-19, participant or staff scheduling constraints, lack of interest in participation, change of contact information, participants being busy with other service needs when they return to clinics (e.g., labs drawn, pharmacist visit, psychiatry visit, social service needs). There were no statistically significant differences on key analytic variables between participants with and without a follow-up assessment. A minority of participants completed the assessment in Spanish (*N* = 28, 11.0%) or Haitian Creole (*N* = 1, 0.4%). Measures that were not already available in Spanish and Haitian Creole were forward- and back-translated by research staff with native fluency in the target language [47]. HIV care related biological outcomes, including HIV RNA viral load and CD4 count, were extracted from participants’ medical records at the clinic and linked to interview data. The University of Miami Institutional Review Board reviewed and approved all study procedures (Protocol Number: 20160911).

### Measures

#### Sociodemographic characteristics

Participants reported their age, racial identity, ethnicity, nativity, sex assigned at birth, gender identity, sexual orientation, educational attainment, employment status, and relationship status at the baseline assessment. These data were used to characterize the sample and are presented in Table 1.

**Table 1:** Participant characteristics

Variable	*N*	%	Variable	*N*	%
Sex assigned at birth			Lifetime trauma types (BTQ) continued		
Male	125	49.0	Serious injury	35	13.7
Female	130	51.0	Violent death of loved one	78	30.6
Gender identity			Witnessing killing or injury	111	43.7
Man	123	48.2	Baseline adherence item 1 (missed doses)		
Woman	129	50.6	0	163	63.9
Trans woman	2	0.8	1	30	11.8
Both man and woman	1	0.4	2	28	11.0
Sexual orientation			> 2	34	13.3
Heterosexual	197	77.3	Baseline adherence item 2 (quality)		
Gay or lesbian	36	14.1	Excellent	171	67.1
Bisexual	17	6.7	Very good	37	14.5
Asexual	1	0.4	Good	24	9.4
Pansexual	1	0.4	Fair or less than fair	22	8.6
Educational attainment			Baseline adherence item 3 (frequency)		
Less than high school	82	32.2	Always	193	75.7
High school graduate	111	43.5	Almost always	31	12.2
Some college	35	13.7	Usually or less than usually	21	8.2
College graduate or more	27	10.6	Follow-up adherence item 1 (missed doses)		
Employment status			0	183	71.8
Employed full time	34	13.3	1	26	10.2
Employed part time	35	13.7	2	25	9.8
Full or part time school	15	5.9	> 2	21	8.2
On disability	144	57.3	Follow-up adherence item 2 (quality)		
Retired	6	2.4	Excellent	174	69.6
Racial and ethnic identity			Very good	37	14.8
Black	192	75.3	Good	25	10.0
White	59	23.1	Fair or less than fair	14	5.6
Multiracial	2	0.8	Follow-up adherence item 3 (frequency)		
Hispanic/Latine	61	23.9	Always	192	76.8
Relationship status			Almost always	43	17.2
Single (never married)	135	52.9	Usually or less than usually	15	6.0
Married/cohabiting	59	23.1			
Non-cohabiting relationship	17	6.7	Variable	M	SD
Divorced or separated	32	12.5	Age	50.98	11.02
Widowed	11	4.3	Lifetime trauma count (BTQ)	2.62	1.83
Lifetime trauma types (BTQ)			Depression symptom severity (PHQ-9)	4.77	5.20
War zone/combat	5	2.0	Anxiety severity (thermometer)	3.40	3.27
Serious accident	84	32.9	PTSD symptom severity (PC-PTSD-4)	1.04	1.42
Natural/technological disaster	153	60.0	Log HIV RNA viral load	1.45	1.17
Life-threatening illness	48	18.8	CD4 count	656.16	326.32
Childhood physical abuse	39	15.3			
Physical assault	55	21.7			
Sexual assault	61	23.9			

#### Lifetime trauma exposure

The Brief Trauma Questionnaire (BTQ) [48] is a self-report scale designed to assess lifetime exposure to traumatic events that may meet “criterion A” for posttraumatic stress disorder (PTSD), as defined by the Diagnostic and Statistical Manual of Mental Disorders (DSM–5-TR) [3]. These events include direct exposure to death, threatened death, actual or threatened serious injury, or actual or threatened sexual violence. Participants responded with “yes” or “no” to indicate if they had experienced each event, and a total score was computed for each participant to represent the number of different types of traumatic events they had experienced over the lifetime up to the baseline assessment. The BTQ has demonstrated moderate to high reliability and validity in prior samples, with interrater reliability kappa coefficients ranging from 0.74 to 1.00 for all items except those asking about illness (0.69) and other life-threatening events (0.60) [49].

#### Baseline depression symptoms

The 9-item Patient Health Questionnaire (PHQ-9) [50] was used to assess the presence and frequency of depressive symptoms over the prior two weeks, in line with DSM-5-TR criteria [3]. Each item is rated on a four-point scale ranging from 0 (*not at all*) to 3 (*nearly every day*). A total score was computed for each participant, with higher scores indicating more severe depressive symptoms at the baseline assessment. Spanish and Haitian Creole versions of the measure have been previously validated [51, 52]. Internal consistency reliability for the PHQ-9 in the current sample was a Cronbach’s α of 0.83.

#### Baseline anxiety

The Anxiety Thermometer, a 10-point visual analog scale, was used to assess the severity of self-rated anxiety in the past week at the baseline assessment. This measure was created by the National Comprehensive Cancer Network and has been widely used in medical settings to examine state anxiety [53]. The scale has shown validity and reproducibility in prior samples, with pre- and post-examination correlation coefficients between 0.60 and 0.78 [54].

#### Baseline posttraumatic stress symptoms

The 4-item Primary Care Posttraumatic Stress Disorder Screen (PC-PTSD-4) [55] is a brief measure designed to identify individuals with probable PTSD in primary care settings. Participants responded with “yes” or “no” to four items mapping onto unique posttraumatic stress disorder symptom clusters, including intrusions, avoidance, negative changes in thinking and mood, and changes in physical and emotional reactions [3]. A total score ranging from 0 to 4 was computed for each participant, with higher scores indicating more severe posttraumatic stress symptoms at the baseline assessment. This scale’s internal consistency reliability in the current sample was Cronbach’s α of 0.83.

#### Follow-up antiretroviral adherence

The 3-item self-report Wilson adherence measure [56] was used to assess antiretroviral adherence at follow-up. This measure assesses adherence behaviors over the past 30 days. Item 1 asks participants to report the number of days they missed at least one dose of their HIV medication in the past month. Item 2 asks participants to rate their performance in taking their HIV medication as prescribed, using response options including *very poor*,* poor*,* fair*,* good*,* very good*,* excellent*. Item 3 asks participants how often they took their HIV medicines as prescribed, using response options including *never*,* rarely*,* sometimes*,* usually*,* almost always*, and *always.*

#### Follow-up HIV RNA viral load and CD4 count

Participant HIV RNA viral load and CD4 count lab results closest in date to the follow-up assessment (M = 4.7 months apart) were extracted from participants’ medical record and linked with the psychosocial interview data.

### Analyses

Data screening and preparation were conducted in IBM SPSS Statistics, Version 27.0. Missingness rates in the analytic sample were low for all variables (< 2.6%) due to the interview-based administration of the measures and real-time quality assurance monitoring. Given the low missingness and to facilitate use of a more appropriate estimator for the type of data we observed (details below), we excluded missing cases pairwise. Our analytic sample size is consistent with other structural equation modeling studies with similarly complex models [57–59] and is considered adequate per standard guidelines in the methodological literature [60–62]. Data was screened for outliers and multivariate normality. To address convergence issues in Mplus, HIV RNA viral load was log-10-transformed and CD4 count was divided by 1000 to reduce variance. We retransformed CD4 count to ease the interpretation of the direct effects. Descriptive statistics for sociodemographic characteristics and observed variables of interest were calculated to characterize the sample.

Structural equation models were estimated in Mplus version 8.1 [63] using weighted least squares with probit link function and delta parameterization given the scaling of the indicators. We pursued model building incrementally, beginning with confirmatory factor analysis to fit latent factor models prior to embedding structural relationships to estimate path coefficients. We constructed a latent factor of adherence indicated by the three items of the adherence measure [56] to improve measurement reliability in the context of noted biases in self-report measures of medication adherence [64, 65]. Prior to fitting the latent adherence factor, we reviewed response frequencies for the three items and re-categorized responses to reduce categories with too few responses while retaining as much nuance as possible. For item 1, we retained categories 0, 1, and 2, but grouped all responses of more than 2 days, which had low endorsement (*N* = 21). For item 2, we retained categories *excellent*, *very good*, and *good*, but grouped all responses of *fair* or less than *fair* (*N* = 14). For item 3, we retained categories *always* and *almost always* but grouped all responses of *usually* or less than *usually* (*N* = 15). Additionally, we constructed a latent factor of posttraumatic psychological distress indicated by depression, anxiety, and posttraumatic stress symptoms. This approach enabled us to consider diverse manifestations of posttraumatic psychological distress in line with prior epidemiological evidence on the consequences of trauma exposure [31, 66–68], and it facilitated improved measurement of posttraumatic distress beyond the narrow scope of the PC-PTSD-4, which includes four dichotomous items. Latent factors of posttraumatic distress are both etiologically coherent, as evidenced by genetic association studies of shared risk across disorders [69, 70], and consistent with prior literature in this area [71, 72].

**Fig. 1:**
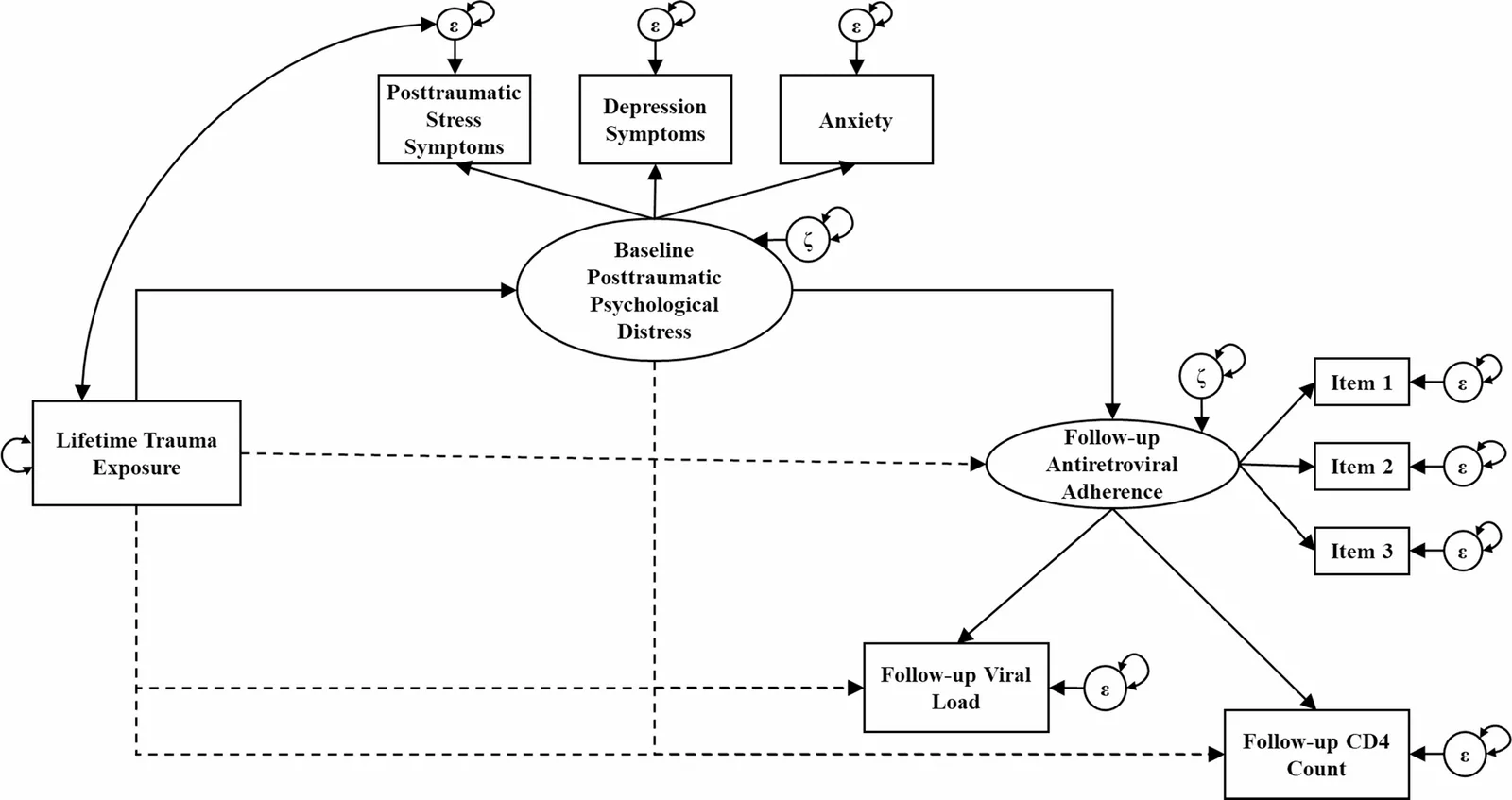
Estimated structural regression model of HIV-related health outcomes. †Rectangles depict observed variables; ovals depict latent variables; straight arrows depict direct effects; curved arrows depict variances and covariances; small circles depict errors and disturbances; solid lines depict significant parameter estimates; dashed lines depict nonsignificant parameter estimates

We then embedded these latent factor models within the full structural regression model, which is depicted in Fig. 1. Specifically, we examined (1) the direct effects of lifetime trauma exposure (assessed at baseline) on baseline posttraumatic psychological distress, and follow-up medication adherence, HIV RNA viral load, and CD4 count; (2) the direct effects of baseline posttraumatic psychological distress on medication adherence, log HIV RNA viral load, and CD4 count, controlling for lifetime trauma exposure; and (3) the associations among adherence, log HIV RNA viral load, and CD4 count, controlling for lifetime trauma exposure and posttraumatic psychological distress. We also assessed whether (4) lifetime trauma exposure influenced adherence, log HIV RNA viral load, and CD4 count indirectly via baseline posttraumatic psychological distress. Although exposure and distress were measured concurrently due to the limited number of assessment waves, we interpret indirect effects from trauma exposure through posttraumatic distress as a mediator given the implied temporal ordering in their measurement (lifetime exposure to trauma, current experiences of distress), consistent with prior studies in this area [73–75].

We fit an initial model that demonstrated poor fit (χ^2^ = 42.872, *p* = 0.0021; RMSEA = 0.067 (90% CI [0.039, 0.095]). Following review of normalized residuals and modification indices in the initial model, we pursued a post-hoc model modification to covary the posttraumatic stress symptom indicator residual term with trauma exposure [76]. This post-hoc model modification was both theoretically reasonable and empirically permitted the estimation of the latent distress factor and achieving model fit to our data. Theoretically, this residual covariance helped to account for the unique etiological relationship between trauma and posttraumatic stress symptoms, above and beyond depression and anxiety, given that PTSD can only occur in the context of trauma exposure, whereas depression and anxiety can have other causes [77]. Empirically, posttraumatic stress symptoms and trauma exposure were significantly related in our model even after accounting for the direct effect of trauma exposure on the posttraumatic distress factor (standardized estimate = 0.395, *p* < 0.001). This modification is consistent with standard procedures in the structural equation modeling literature [76] and has been previously used in similar studies [see e.g., 71]. Although the initial model did not have good fit, review of the output revealed generally consistent parameter estimates with our final fitting model.

We evaluated global and local model fit consistent with conventional index thresholds appropriate for weighted least squares estimation [62], including a non-significant Chi-square, RMSEA < 0.06, CFI > 0.95, SRMR < 0.08, modification indices < 10.0, and normalized residuals < |2.0| [61, 76, 78]. Structural parameters of interest included unstandardized effect estimates, confidence intervals, p values, and effect sizes (R^2^). Significance of the indirect effects was assessed via bootstrapped confidence intervals rather than p values [79].

In the results, we present a parsimonious model that clearly specifies the causal paths we were interested in, which were previously reviewed. However, we completed sensitivity analyses of our effects to examine whether our findings were robust to sample idiosyncrasies, given our reliance on a convenience sample recruited in a clinical setting. First, we assessed whether relevant socioeconomic (educational attainment, annual income) and psychosocial (housing instability, substance use) covariates confounded the relationships among trauma, distress, and HIV outcomes. Our sensitivity analysis found that these covariates were significantly related to distress, but not the HIV outcomes, and they did not change the observed pattern of results. Second, we assessed whether our sample’s ethnic, racial, and linguistic diversity may have influenced the observed relationships. Introducing interaction terms for these identity categories resulted in poorly fitting models, which may be the results of the moderators not being relevant in this sample, or, more likely, due to the introduced complexity of the model in our small sample size. Without formal invariance testing, which our limited sample size did not permit, it is unclear whether different groups of participants in our study responded invariantly to the self-report measures, which is particularly important to examine for Spanish- and Haitian Creole-speaking participants. However, rerunning our analysis excluding these participants yielded no differences.

## Results

Participants were between the ages of 23 and 80 (M = 50.98, SD = 11.02). The sample was primarily Black (*N* = 191, 74.9%), followed by Hispanic/Latine (*N* = 56, 22.0%), and non-Hispanic White (*N* = 7, 2.7%). Most participants endorsed high school as their highest level of educational attainment (*N* = 193, 75.7%) and were on disability at baseline (*N* = 144, 57.3%). The survey was completed in English (*N* = 220, 86.3%), Spanish (*N* = 28, 11.0%), both English and Spanish (*N* = 5, 2.0%), and Haitian Creole (*N* = 1, 0.4%). Participants were exposed to 2.62 out of 10 assessed potentially traumatic event types on average (SD = 1.83), with 88.2% endorsing a prior history of trauma exposure (i.e., at least one potentially traumatic event type, *N* = 225). Overall, participants reported high levels of adherence, with 1 day of nonadherence in the past month on average (SD = 3.55). Most participants (*N* = 226, 88.6%) had a suppressed viral load (i.e., ≤ 200 copies/mL) at follow-up, and the average CD4 count was in the clinically normative/healthy range (M = 656.16, SD = 326.32) [80]. Complete participant characteristics are presented in Table 1.

The final structural equation model (Fig. 1) evidenced good fit to the data. Global model fit was acceptable, χ^2^(19, *N* = 255) = 20.322, *p* = 0.375; RMSEA = 0.017 (90% CI = [0.000, 0.058]); CFI = 0.999; SRMR = 0.026. The indicators had acceptable loadings onto the factors of posttraumatic psychological distress and adherence (standardized loadings > 0.4; *p* < 0.001, see Table 2). Normalized residuals were all below 2.0, and no modification indices exceeded 10.0.

**Table 2:** Factor loadings in measurement models of posttraumatic psychological distress and medication adherence

Indicator	Standardized estimate	*P* value	Lower confidence bound	Upper confidence bound
Posttraumatic psychological distress factor				
Depression	0.815	< 0.001	0.669	0.961
Anxiety	0.713	< 0.001	0.566	0.860
Posttraumatic Stress	0.454	< 0.001	0.327	0.582
Medication adherence factor				
Item 1	0.941	< 0.001	0.904	0.977
Item 2	0.923	< 0.001	0.887	0.959
Item 3	0.956	< 0.001	0.922	0.991

Direct and indirect effects of the structural model are summarized in Table 3. Lifetime exposure to trauma was significantly associated with psychological distress at baseline. Specifically, each additional type of traumatic event endorsed was associated with a 0.281-unit increase (β = 0.22, a small-to-medium effect) in psychological distress score, *p* = 0.004, 95% CI [0.089, 0.474]. Psychological distress at baseline predicted medication adherence at follow-up, such that per unit increase in psychological distress, adherence ratings decreased by 0.070 points (β = -0.17, a small effect), controlling for lifetime trauma exposure, *p* = 0.033, 95% CI [-0.135, -0.006]. Medication adherence was significantly inversely associated with log HIV RNA viral load, such that lower adherence was associated with higher viral load (b = -0.477, *p* < 0.001, 95% CI [-0.642, -0.313], β = -0.38, a medium effect). Medication adherence was significantly directly associated with CD4 count, such that lower adherence was associated with decreased CD4 count (b = 84.754, *p* < 0.001, 95% CI [37.861, 131.645], β = 0.24, a small-to-medium effect). Trauma exposure had no significant direct or indirect effects on medication adherence, HIV RNA viral load, or CD4 count, in the presence of the other variables. Psychological distress had no significant direct effects on HIV RNA viral load, or CD4 count, in the presence of the other variables.

**Table 3:** Direct and indirect effect parameter estimates of structural regression model

Criterion	Predictor	Unstandardized estimate	Standard error	*p* value	Lower bound	Upper bound	Standardized estimate	*R* ^2^
Direct effects								
Baseline psychological distress	Lifetime trauma exposure	0.281	0.098	0.004	0.089	0.474	0.221	0.049
Follow-up antiretroviral adherence	Lifetime trauma exposure	0.040	0.036	0.264	−0.112	0.031	−0.079	0.043
	Baseline psychological distress	−0.070	0.033	0.033	−0.135	−0.006	−0.174	
Follow-up log HIV RNA viral load	Lifetime trauma exposure	−0.026	0.045	0.559	−0.115	0.062	−0.041	0.149
	Baseline psychological distress	0.013	0.032	0.677	−0.049	0.075	0.026	
	Follow-up antiretroviral adherence	−0.477	0.084	< 0.001	−0.642	−0.313	−0.384	
Follow-up CD4 count	Lifetime trauma exposure	−0.906	12.239	0.941	−24.895	23.083	−0.005	0.058
	Baseline psychological distress	8.224	9.520	0.388	−10.435	26.882	0.059	
	Follow-up antiretroviral adherence	84.754	23.810	< 0.001	38.087	131.420	0.244	
Indirect effects								
Follow-up antiretroviral adherence	Lifetime trauma exposure	−0.020	0.012	--	−0.045	0.000	−0.038	0.043
Follow-up log HIV RNA viral load	Lifetime trauma exposure	0.009	0.007	--	0.000	0.025	0.015	0.149
Follow-up CD4 count/1000	Lifetime trauma exposure	−0.002	0.001	--	−0.005	0.000	−0.009	0.058

## Discussion

The current study estimated a structural equation model among PLWH receiving care in a real-world HIV treatment setting in a U.S. HIV epicenter to differentiate the effects of trauma exposure and posttraumatic distress on HIV-related behavioral and biological outcomes longitudinally. When considering both exposure to and consequences of trauma together, we found that only posttraumatic psychological distress, but not trauma exposure, predicted lower HIV medication adherence at follow-up, which was in turn associated with higher HIV RNA viral load and lower CD4 count. These effects were established longitudinally, at approximately one year later, in a sample with relatively high engagement in HIV care.

The lack of direct effects from trauma exposure or posttraumatic distress on HIV RNA viral load and CD4 count outcomes may be understood in the current context of highly effective and relatively permissive antiretroviral regimens. Specifically, we observed a small effect size on adherence, which may not be large enough to effect differences on neuroimmune targets. This is particularly likely in the case of our highly adherent sample, given that recent evidence indicates that as low as 75% medication adherence may be sufficient to sustain viral suppression [81]. Additionally, effects on neuroimmune targets typically require a longer time to accumulate and a longer follow-up period to observe [see e.g., 82].

Our findings are broadly consistent with and build on prior literature in this area. In line with longstanding evidence that “trauma” is implicated in HIV care, our findings validate that trauma is highly common among PLWH, and that experiences related to trauma can undermine HIV-related health outcomes, even in a highly adherent sample [1, 4–8]. By including both variables in the same model, we demonstrated that psychological distress uniquely predicts HIV medication adherence longitudinally after accounting for lifetime trauma exposure. Conversely, when baseline psychological distress was accounted for, lifetime trauma exposure was no longer associated with HIV outcomes at follow-up. This suggests that the effect of trauma on HIV health may operate specifically through posttraumatic distress. We extended prior work by leveraging a longitudinal sample, in which we linked distress to persistent decrements in adherence one year later. Additionally, leveraging structural equation modeling methods, we were able to consider multifaceted manifestations of posttraumatic psychological distress, including symptoms of depression, anxiety, and posttraumatic stress disorder. Overall, these findings show that even in the era of highly efficacious and relatively permissive HIV medications, subtle subjective differences in adherence related to psychosocial concerns continue to be associated with relevant biological outcomes.

Given that trauma exposure appears to be the norm rather than the exception in this population, trauma-informed care principles may be integral to any HIV care setting. In recognition that health care interactions can be re-traumatizing, trauma-informed care is an organizational and clinical approach to service delivery that emphasizes safety, empowerment, and healing for persons impacted by trauma. For example, trauma-informed HIV care practices may include educating both clinical and administrative staff about the frequency, signs and symptoms, and consequences of trauma exposure, creating safe environments for patients, such as by making sure they have easy access to exits, obtaining consent prior to physical evaluations and allowing patients to forego physical exams, and emphasizing patient participation in treatment decision making [83–85]. Prior trauma exposure was significantly associated with distress in our analysis, indicating that HIV care settings may become more responsive to the needs of their patients by initiating universal screening for trauma and adapting environmental and interpersonal contacts within the care setting. Recent evidence from real-world implementation efforts of trauma-informed care in HIV treatment settings has identified both successes (e.g., universal trauma screening and systematized referral practices within trauma-informed health settings) and persistent barriers (e.g., lack of training and relative priority against other service needs) to sustainment [86–89]. In other words, the absence of an independent direct effect of trauma exposure in the presence of distress does not indicate that traumatic events are themselves negligible.

At the same time, patients who experienced posttraumatic psychological distress in our study represented a priority group requiring a higher level of intervention. Therefore, our findings highlight the need for a dual approach, including universal trauma-informed care for all patients given high levels of trauma exposure, as well as focused delivery of clinical interventions to those patients who also experience distress following trauma. Integrated behavioral health care is a model of care delivery that employs multidisciplinary treatment teams that can address both physical and mental health concerns within the same setting, with the aim of improving screening and identification of interfering psychosocial concerns and physical-mental healthcare coordination [90]. Integrated behavioral health services are typically delivered in primary care settings that have the capacity to integrate routine mental and behavioral health screening, brief embedded behavioral health interventions, and psychiatric diagnosis and pharmacological treatment for mood, anxiety, trauma, and other concerns commonly presenting within medical settings [91, 92]. Although not thoroughly tested in HIV care settings [93, 94], brief, evidence-based trauma treatment has been shown to be acceptable and effective when embedded within other primary care settings [95, 96].

### Limitations

Study findings and implications should be considered in the context of methodological limitations. Given that our study leveraged a participant population accessing a real-world treatment setting, it was restricted to persons relatively well engaged in HIV care. This may limit the generalizability of our findings, since those PLWH most impacted by trauma and associated distress are also more likely to disengage from care. As a result, the effects of trauma and distress on HIV health outcomes may be even more pronounced in the general population of PLWH that includes lower levels of care engagement. Relatedly, our sample was representative of the clinic’s patient population, which is demographically and culturally diverse. Replications in other settings, especially those supported by culturally validated measures of trauma and distress [97, 98], will be helpful to strengthen the literature on the effects of trauma in HIV health.

In our use of measures commonly available within primary care settings, we relied primarily on brief self-report measures. Although these measures are preferred in standard clinical practice since they support efficient and low-burden assessment and monitoring of concerns, they pose challenges in measurement validity. Regarding the measurement of trauma exposure, we only assessed how many different types of traumatic events participants had experienced, limiting important insights regarding frequency (e.g., total count of events beyond event types), chronicity, timing (e.g., occurring during critical developmental periods), or severity of exposure to traumatic events, all of which may be necessary components to fully specify the trauma exposure construct and meaningfully estimate its effects on relevant outcomes [99, 100]. For example, a participant with one exposure to a serious hurricane received the same BTQ score as a participant with chronic and repeated instances of childhood sexual abuse, which limits the validity of our trauma exposure measure. Further, environmental trauma exposures were overrepresented in our sample, but are typically associated with less severe and chronic posttraumatic trajectories compared to interpersonal victimization traumas [101], which could have affected our findings on the effects of trauma exposure. Future studies with wider representation of trauma types could help to provide evidence of generality of our findings. Regarding the measurement of distress, we had access to limited information based on brief symptom screening measures, which we tried to mitigate through our use of latent variable modeling. However, clinician-administered measures of psychological distress may help to minimize recall, desirability, and other self-report biases in future studies [102]. Regarding the measurement of adherence, our study utilized a validated self-report measure, improved measurement reliability by using latent variable modeling, and associated this self-report measure with objective markers of HIV health through patient medical records. Nevertheless, highly sophisticated measures to objectively monitor adherence are available for research use and may be used to more rigorously examine the effects of trauma on HIV health related behaviors [103].

Finally, our study findings are limited by the concurrent measurement of trauma and distress at the baseline assessment. Although this is a reasonable approach given the temporal ordering implied in the measures themselves, it prevents a formal test of causal mediation and increases the risk of overadjustment, which may have biased the estimates of the effects of trauma exposure towards the null. Future studies with additional assessment waves could better temporally separate the measurement of the predictor and mediator and thus provide stronger evidence in support of the mechanistic effects of trauma.

## Conclusion

Leveraging data from a real-world HIV treatment setting, we aimed to examine the longitudinal effects of trauma on HIV-related behavioral and biological health outcomes. First, we found that trauma exposure was very common in this setting, with only one in 10 PLWH denying any lifetime trauma exposure. Second, we found that the added presence of posttraumatic psychological distress predicted poorer HIV medication adherence among PLWH exposed to trauma. These findings contribute to improved clarity and scientific precision in the HIV literature. Additionally, findings indicate that there is an opportunity to improve both psychological and HIV-related health outcomes by integrating mental health screening and intervention services within primary care clinics.
